# The Histological and Immunohistochemical Aspects of Bile Reflux in Patients with Gastroesophageal Reflux Disease

**DOI:** 10.1155/2011/905872

**Published:** 2011-07-24

**Authors:** Andreas Nakos, Georgios Kouklakis, Michail Pitiakoudis, Petros Zezos, Eleni Efraimidou, Alexandra Giatromanolaki, Alexandros Polychronidis, Nikolaos Liratzopoulos, Efthimios Sivridis, Konstantinos Simopoulos

**Affiliations:** ^1^Gastrointestinal Endoscopy Unit, Democritus University of Thrace, University General Hospital of Alexandroupolis, Dragana, 68100 Alexandroupolis, Greece; ^2^2nd Department of Surgery, Democritus University of Thrace, University General Hospital of Alexandroupolis, Dragana, 68100 Alexandroupolis, Greece; ^3^1st Department of Surgery, Democritus University of Thrace, University General Hospital of Alexandroupolis, Dragana, 68100 Alexandroupolis, Greece; ^4^Pathology Department, Democritus University of Thrace, University General Hospital of Alexandroupolis, Dragana, 68100 Alexandroupolis, Greece

## Abstract

*Introduction*. The pathogenesis of GERD is strongly related with mixed acid and bile reflux. Benign and malignant esophageal and gastric lesions have been associated with synergetic activity between those parameters. Bile reflux causes reactive gastropathy evaluated with Bile Reflux Index (BRI). The aim was to investigate if the sequence: *bile reflux-intestinal metaplasia-GERD-esophagitis,* is associated with apoptotic/oncogenetic disturbances. *Materials/Methods*. Fifteen asymptomatic subjects and 53 GERD patients underwent gastroscopy with biopsies. The specimens examined histologically and immunohistochemically for p53, Ki-67, Bax, and Bcl-2. *Results*. Elevated BRI score detected in 47% (25/53) of patients with GERD and in 13% (2/15) of controls (*P* = 0.02). Severe esophageal lesions were significantly more common in BRI (+) patients (14/25) compared to BRI (−) ones (*P* = 0.0049). Immunohistochemical analysis did not show associations between BRI score and biomarker expression. *Conclusions*. Bile reflux gastropathy is associated with GERD severity, but not with oncogene expression or apoptotic discrepancies of the upper GI mucosa.

## 1. Introduction

The gastroesophageal reflux disease (GERD) represents one of the most common gastrointestinal disorders, especially in the western countries [1, 2]. It is the result of the exposure of the esophageal mucosa to acidic gastric juice and/or bile-containing duodenal refluxates via an incompetent lower esophageal sphincter. The acid and bile exposure times, as demonstrated by 24 h monitoring methodologies, greatly overlap among the cases of GERD with diverse endoscopic findings ranging from normal mucosa (nonerosive reflux disease (NERD)) to severe reflux oesophagitis, Barrett's esophagus, and complications such as strictures or ulcers [3–6].

Although in the past the pathogenesis of GERD was strongly associated with the gastric acid, during the last two decades the role of mixed reflux has been increasingly investigated. The synergetic activity between acid and bile has largely been implicated in the clinical spectrum of GERD and methods such as Bilitec 2000 and the multichannel intraluminal impedance are nowadays widely used towards the thorough evaluation of the disorder [7]. The highest esophageal exposure to bile has been observed in patients with Barrett's dysplasia and esophageal adenocarcinoma. However, it has also been associated with erosive oesophagitis, Barrett's esophagus without dysplasia, and intestinal metaplasia of the gastric antrum [8–15]. 

The passage of duodenal contents through the stomach produces consistent histological changes in the gastric mucosa (reactive gastropathy) with antral intestinal metaplasia being one of the most prominent histological characteristics. Bile reflux is also positively associated with the severity of glandular atrophy, chronic inflammation, lamina propria edema, and foveolar hyperplasia. As a result, a histological index, the *Bile Reflux Index *(*BRI score*), was derived to measure the severity of bile-induced histological changes in the gastric antrum mucosa [16].

One of the most serious complications of GERD is Barrett's esophagus. It represents a premalignant condition of the distal esophagus in which squamous epithelial cells are replaced by metaplastic intestinal-like columnar epithelium that contains goblet cells. Apoptosis seems to maintain esophageal tissue homeostasis, which is regulated by p53, and is gradually lost in the metaplasia-dysplasia-carcinoma sequence of Barrett's esophagus [17]. Similarly, apoptosis is involved in gastric epithelial homeostasis and integrity since its deregulation is associated with the occurrence of lesions such as atrophic gastritis, peptic ulcers, intestinal metaplasia, and gastric malignancies [18].

In a previous study, we reported that the presence of bile reflux gastropathy in patients with GERD was associated with more severe disease [19]. The aim of this study was to investigate if the reflux of bile into the stomach and the esophagus could be associated with apoptotic disorders and abnormal oncogene expression. For this purpose, the mucosal expression of the biomarkers p53, Ki-67, Bax, and Bcl-2 was immunochistochemically examined.

## 2. Materials and Methods

Fifty-three patients (42 men and 11 women, mean age 33.3 years, range 18–76) with at least 12-week total duration of reflux symptoms and a frequency of three times a week/four hours a day during the last month, underwent upper gastrointestinal (GI) endoscopy, after completing a standardized questionnaire with demographic and clinical details. 

Exclusion criteria were (1) previous foregut surgery; (2) treatment with nonsteroid anti-inflammatory or antiplatelet drugs; (3) previous history of *Helicobacter pylori* (H. p.) eradication; (4) portal hypertensive gastropathy or varices (gastric or esophageal); (5) corrosive oesophagitis by a toxicant; (6) alcohol abuse; (7) scleroderma; (8) active ulcer disease; (9) neoplasia or Crohn's disease of the upper GI tract; (10) systematic use of PPIs. 

Standard complete upper GI endoscopy was performed with the Fujinon 250 HR gastroscope by the same endoscopist (A. Nakos) after a 12-hour fasting period. Reflux oesophagitis was graded from A (least severe) to D (most severe), according to the Los Angeles classification system, whereas hiatal hernia was considered if gastric folds were extending ≥3 cm above the diaphragmatic hiatus. After detection of the lesions, two biopsies were taken from gastric antrum, two from gastric body and cardia, and two from the esophagus. The biopsies from the esophagus were taken from its lowest 5 cm, each specimen from the right and left esophageal wall, respectively. In case of erosions, biopsies were taken besides them. If Barrett's esophagus was present, both columnar and squamous epithelium were biopsied. Control biopsies obtained from 15 healthy control (HC) subjects (9 men and 6 women, mean age 40.4 years, range 23–67) were without symptoms of GERD and no endoscopic evidence of erosive oesophagitis or Barrett's. Nine of the healthy controls underwent endoscopy to exclude gastric pathology and the other 6 were healthy volunteers. Informed consent was obtained from each patient and healthy control volunteer, and the study was approved by the local research ethics committee.

All biopsy specimens were fixed in 10% neutral formalin for at least 3 hours, processed, sectioned at 4 *μ*m, and stained by haematoxylin-eosin, alcian blue/periodic acid-Schiff (AB/PAS), and Giemsa. Histological examination, based on the updated Sydney system included the investigation for the presence of acute and chronic inflammation, edema, and atrophy, *H. pylori* status, intestinal metaplasia, dysplasia, and BE. Each finding was graded on a 0–3 scale (absent, mild, moderate, and marked, resp.) and the BRI score was calculated in antral biopsies according to the formula: (7 × edema) + (3 × IM) + (4 × chronic inflammation) − (6 × *H. pylori*). Bile Reflux Index values above 14 are considered abnormal and give the best prediction of a raised concentration of bile acid into the gastric juice [16]. 

Apart from the routine histological evaluation and the BRI score calculation, four slides carrying two-sections each were immunohistochemically examined for the p53, Ki-67, Bax, and Bcl-2 expression (Biogenex Laboratories, Inc., San Ramon, Calif, USA). Bax and Bcl-2 were checked in columnar epithelium only, which means in gastric mucosa and in Barrett's esophagus, whereas p53 and Ki-67 were examined in all specimens. According to the percentages of the stained epithelial cells, the immunohistological sections were scored and graded as follows: 0 = absence of staining, 1 = staining up to 5%, 2 = staining >5%–10%, 3 = staining >10%–25%, 4 = staining >25%–50%, and 5 = staining >51%. The biopsies were assessed by one experienced GI pathologist (E. Sivridis) who was unaware of the clinical condition.

For the statistical analysis, continuous data are presented as mean values along with 95% CI and categorical data are presented as proportions. The patients and the HCs with BRI score >14 were characterized as *bile reflux positive* (BRI +) and those with BRI score ≤14 as *bile reflux negative *(BRI −). In GERD patients, the two groups were at first compared for differences concerning demographic data, clinical parameters, endoscopic, and histological findings. Both groups were subsequently evaluated for differences in immunohistochemical characteristics of the gastric and esophageal mucosa, regarding the investigated biomarkers. Correlation and logistic regression analyses were also performed in order to assess which demographic, clinical, endoscopic, or histological parameters had significant influence on the severity of immunohistochemical expression of the biomarkers in gastric and esophageal mucosa. In these analyses we assumed immunohistochemical staining grade 0–3 as score 0 and staining grade 4-5 as score 1. Furthermore, similar analyses were performed in the HC group, and differences between GERD patients and HCs were also investigated. The level of statistical significance was set at *P* < 0.05.

## 3. Results

Demographic, clinical, endoscopic and histological data of GERD patients and healthy controls are shown in Tables 1 and 2. The BRI score was elevated (>14) in 47.1% (25/53) of the GERD patients (bile reflux positive group) and in 13.3% (2/15) of the control subjects (Fisher's exact two-tailed test; *P* = 0.02). Additionally, GERD patients had lower BMI score (Table 1), whereas gastritis, presence of bile into the stomach, atrophy and enteric metaplasia were more common in gastric endoscopy and histology in GERD patients as compared to healthy controls (Table 2).

Demographic, clinical, endoscopic, and histological data of BRI-positive and BRI-negative GERD patients are shown in Tables 3 and 4. Overall, severe esophageal lesions (oesophagitis ≥ grade B and Barrett's) were significantly more common in BRI (+) GERD patients (14/25) compared to BRI (−) (5/28) (56% versus 17.9%, respectively, Fisher's exact two-tailed test; *P* = 0.0049). In 1 BRI (+) GERD patient, both Barrett's esophagus and severe oesophagitis were simultaneously observed, while 2 patients had only Barrett's esophagus without esophagitis. Histological classification of the esophagitis was not used. In the vast majority of cases, the inflammatory infiltration was mild and not correlated with the endoscopic appearance. Its presence in some control cases was also minimal (data not presented). In patients with Barrett's esophagus, the typical endoscopic findings were also histologically confirmed in all cases. None of them had dysplastic epithelium. Furthermore, 13 out of 18 patients with GERD severe esophageal lesions had an elevated Bile Reflux Index score (72,2%).

**Figure 1:**
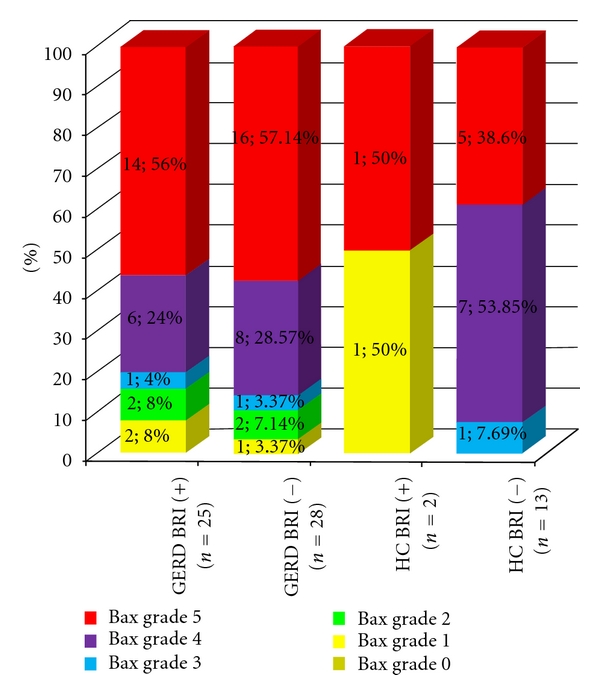
Bax expression in gastric mucosa in healthy controls (HCs) and GERD patients, in relation to BRI status. Grouped data are presented in 100% display stacked columns for each grade (*n*; %). According to the percentages of the stained epithelial cells, the immunohistological sections were scored and graded as follows: 0 = absence of staining, 1 = staining up to 5%, 2 = staining >5%–10%, 3 = staining >10%–25%, 4 = staining >25%–50%, and 5 = staining >51%.

On the contrary, the results of the immunohistochemical studies did not differ between the BRI-positive and BRI-negative GERD patients. We found absence of p53 staining in the esophagus in 15 BRI (+) and 21 BRI (−) patients, *score 1* in 9 BRI (+) and 7 BRI (−) patients, and *score 3* in 1 patient. In the control group, an absence or very low expression of esophageal p53 was detected. There were no significant differences in esophageal mucosa p53 expression between healthy controls and GERD patients, and/or BRI-positive and BRI-negative subjects (Figure 1).

At the stomach, p53 staining was absent in the majority of GERD patients, (in 47 out of 53; 88.6%; 21 BRI (+) and 26 BRI (−) patients, resp.) and *score 1* (≤5%) in 6 out of 53 (11.4%) GERD patients (4 BRI (+) and 2 BRI (−) patients, resp.). In the control group, an absence or very low expression of gastric p53 was also detected. There were no significant differences in gastric antrum mucosa p53 expression between healthy controls and GERD patients, and/or BRI-positive and BRI-negative subjects (Table 5).

Ki-67 staining was present in the esophageal epithelium in almost all tissue samples either from BRI (+) or BRI (−) GERD patients. However, low expression of esophageal Ki-67 expression was detected in the majority of GERD patients, as it also happened in the control group. There were no significant differences in esophageal mucosa Ki-67 expression between healthy controls and GERD patients, and/or BRI-positive and BRI-negative subjects. 

Similarly, Ki-67 in the gastric mucosa was equally expressed between the two GERD groups. On the other hand, low expression was detected in healthy controls, although elevated (*score 4*) expression of Ki-67 was detected in 1 healthy BRI-positive subject. In general, there were no significant differences in esophageal mucosa Ki-67 expression between healthy controls and GERD patients, and/or BRI-positive and BRI-negative subjects (Table 6).

An overexpression of the biomarker Bax that promotes apoptosis was noticed in the gastric mucosa. However, it was not affected by the level of BRI score. Increased expression of Bax, as expected, was also noticed in HCs. There were not significant differences in gastric Bax expression between healthy controls and GERD patients, and/or BRI-positive and BRI-negative subjects (Figure 1).

**Figure 2:**
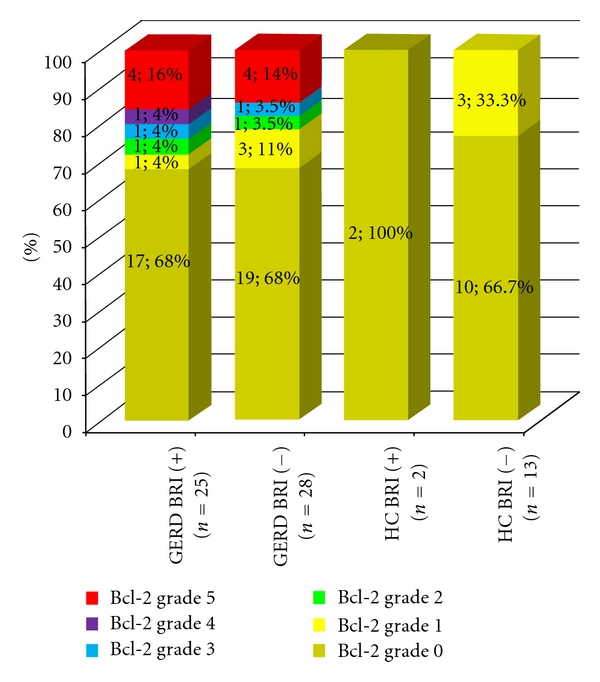
Bcl-2 expression in gastric mucosa in healthy controls (HC) and GERD patients, in relation to BRI status. Grouped data are presented in 100% display stacked columns for each grade (*n*; %). According to the percentages of the stained epithelial cells, the immunohistological sections were scored and graded as follows: 0 = absence of staining, 1 = staining up to 5%, 2 = staining >5%–10%, 3 = staining >10%–25%, 4 = staining >25%–50%, and 5 = staining >51%.

On the other hand, although the antiapoptotic factor Bcl-2 in antral mucosa was absent in 67.9% (36/53) of GERD patients, an overexpression (*score 5*) was detected in 15% (8/53) of them, without any association with the BRI levels (equal expression in both groups). Furthermore, an absence or very low expression of Bcl-2 was detected in the control group. No significant differences in gastric Bcl-2 expression between healthy controls and GERD patients, and/or BRI-positive and BRI-negative subjects were detected (Figure 2). No association was also revealed between the presence of *Helicobacter pylori* and the expression of the biomarkers in the gastric mucosa (data not shown).

Overall, correlation and regression analyses did not reveal any significant influence of demographic, clinical, endoscopic, or histological parameters on the severity of immunohistochemical findings in gastric and esophageal mucosa (data not shown).

## 4. Discussion

The gastro-esophageal reflux disease is pathogenetically associated with both acid and bile reflux. The synergism between acid and duodenal contents has been associated with significant esophageal injury and progression of metaplasia to Barrett's esophagus [20–22]. Although the majority of patients with GERD have no esophageal lesions (NERD), a significant proportion develops oesophagitis, whereas Barrett's is diagnosed in about 10% of patients.

The duodeno-gastroesophageal reflux nowadays, is quantitatively evaluated by highly sophisticated and reliable methods such as Bilitec 2000 or the multichannel intraluminal impedance [23]. However, bile reflux is also histologically evaluated according to the bile-induced reactive gastropathy and expressed by the Bile Reflux Index [24].

In a previous study, we used the BRI score and found that about half of GERD patients had histological lesions due to bile reflux into the stomach and that BRI score was associated with the endoscopic severity of the esophageal disorder. Consequently, evaluating the histological evidence of bile reflux in the stomach, we indirectly showed the implication of duodeno-gastroesophageal reflux (DGER) in the pathogenesis of GERD [19].

In this study and in the same group of patients, we evaluated the mucosal expression of tumor and apoptotic markers using immunohistochemical methods. The aim was to investigate possible associations existing between GERD/DGER and the early stages of carcinogenesis. For this purpose, the mucosal expression of the biomarkers p53, Ki-67, Bax, and Bcl-2 was evaluated.

*p53* is a tumor suppressor gene which encodes a nuclear protein that plays an important role in cell cycle control, DNA synthesis and repair, cell differentiation, and apoptosis. Mutant forms of *p53* gene are usually associated with accumulation of the protein in the cell, which can be detected by using specific antibodies [25]. Bcl-2 protein prolongs cell survival and is considered to be a suppressor of apoptosis whereas Bax protein plays a key role in apoptosis by accelerating cell death after an apoptotic stimulus. Ki-67 is a nuclear antigen that is present in proliferating cells and it has been used as an index of cell proliferation [26–29].

Although Barrett's esophagus and esophageal adenocarcinoma have extensively been studied with immunohistochemical methods, there is a lack of immunohistochemical studies for benign disorders such as erosive oesophagitis or NERD in association or not with DGER [30, 31]. On the other hand, the association between DGER, GERD and, carcinogenesis is constantly suggested and in a recent study Shaheen and Ransohoff reported that the risk of esophageal adenocarcinoma is increased fivefold in patients with previously diagnosed oesophagitis. However, most of the adenocarcinomas occurred among patients with Barrett's esophagus [32]. 

Numerous studies and animal models have shown the carcinogenetic effect and mutagenic potential of DGER in the esophageal mucosa. The mechanism is probably indirect, involving the induction of oxidative stress and production of reactive oxygen species that damage DNA. Repeated DNA damage likely increases the mutation rate, including the mutation rate of tumor suppressor genes and oncogenes [33–35].

In the present study, we immunohistochemically demonstrated a minimal oncogene expression, suggesting that the potential risk for esophageal carcinogenesis in patients with GERD is extremely low regardless of bile reflux contribution. Specifically, the very low expression of *p53* in the esophageal specimens, with staining cells up to 5% in 96.10% of patients (52/53), means that the mutation rate of the *p53 *gene, which is considered one of the most serious initial events in tumorigenetic process, was not increased. Even low p53 protein expression was unrelated with the presence of bile reflux gastropathy, the levels of BRI score or the endoscopic severity of the esophageal lesions. Previous studies have demonstrated that positive rates of p53 protein expression in esophageal mucosa were gradually increased from normal to reflux oesophagitis and furthermore to Barrett's metaplasia and dysplasia [36]. 

As it has been mentioned above, the accumulation of p53 protein in the cell is highly suggestive for *p53* gene mutation. However, positive *p53* immunostaining in some tissue samples is related to inactivation of p53 protein by different mechanisms than the mutation of *p53* gene, such as mucosal inflammation. The histological examination of the esophageal biopsies of our patients revealed the presence of mild to moderate chronic inflammation in the majority of them (45/53; 84.9%,Tables 2 and 4) and this phenomenon could be related with the low expression of p53 in our GERD patients. In that case, the low p53 expression in our cases has practically no carcinogenetic effect because it is probably associated with the inflammation and not with mutations [37].

Regarding the Ki-67 cell proliferation marker, we found a very low esophageal expression in 64.1% (45/53) of our patients. Similarly, Feith et al. investigating the malignant degeneration of Barrett's esophagus showed that there was a low expression of Ki-67 in normal squamous epithelium of the esophagus (only 20.4 ± 6.4% of cells were Ki-67-positive) compared to columnar metaplasia (34.9 ± 11.1%), dysplasia (44.8 ± 8.9%), and invasive carcinoma (60.8 ± 17.9%) [38]. We found no statistically significant associations between Ki-67 reactivity either with the BRI positivity or the severity of the esophageal lesions, even in the 6 cases (11.3%) with moderate or high Ki-67 expression (>25% of cells stained).

As it has been detected, the presence of bile reflux gastropathy in our GERD patients was not associated with p53 or Ki-67 expression in the esophageal mucosa. Consequently, bile reflux seems not to change the theory that there is a lack of genetic susceptibility markers for the development and early progression of GERD to Barrett's esophagus and esophageal cancer [39].

In accordance with the esophagus, we found no association between the bile reflux gastropathy and deregulation of apoptosis or oncogene expression in the gastric mucosa. 

The very low expression of p53 in the gastric mucosa in a small number of patients is also suggestive that an accumulation of wild-type protein occurred, probably due to an inflammatory response. Our findings are compatible with Craanen et al.'s study who described an absence of p53 expression in benign gastric lesions [40]. However, many investigators have already supported that *H. pylori* infection is associated with the increased expression of p53 in gastric mucosa [41, 42].

**Figure 3:**
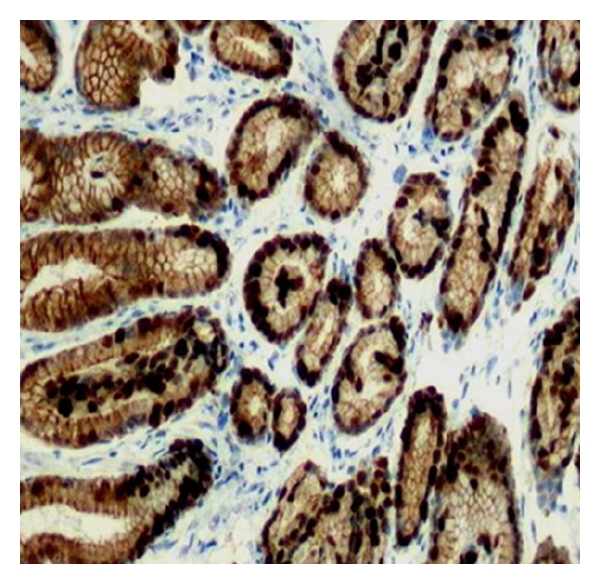
Ki-67 expression in the gastric mucosa (magnification ×100). The immunoreactive cells (dark nuclei) are mainly located at the base of the epithelium.

Furthermore, the high expression of the Bax protein in the gastric mucosa of our patients is indicative that the process of apoptosis is not affected and it plays an important protecting role against carcinogenesis. Similarly, the Ki-67 immunoreactive cells of our patients were mainly localized at the base of the epithelium, which correspond to the naturally occurring proliferative zone (Figure 3).

An overexpression of the Bcl-2 protein at the stomach was detected in 15% of our patients. However, it was not statistically associated either with the bile reflux gastropathy or with the *H. pylori* infection. Moreover, regression analyses did not reveal any significant influence of demographic, clinical, endoscopic, or histological parameters on the severity of immunohistochemical expression the specific antiapoptotic marker in gastric mucosa. However, in contrast with our findings, the significance of Bcl-2 expression at the stomach has been previously reported and found to be implicated in the multistep process, ranging from chronic gastritis to atrophy, intestinal metaplasia, dysplasia, and finally invasive gastric cancer [43].

## 5. Conclusions

To summarize, we could say that our study demonstrated that, although the bile reflux significantly contributes in the pathogenesis of GERD, it has practically no impact on the process of apoptosis or primary carcinogenetic effect in the upper GI mucosa of these patients. However, the fact that the patients of our study are relatively young is indicating a conscious interpretation of the results, especially as far as the immunohistochemistry is concerned. On the other hand, larger series of patients and probably more specific and sensitive methods of evaluation of bile reflux, than histological evidence only, are needed in order to evaluate the significance of our findings.

## Figures and Tables

**Table 1:** Demographic and clinical data in GERD patients and healthy controls.

	GERD patients (*n* = 53)	Healthy controls (*n* = 15)
Gender, *n* (M/F)	42/11	9/6
Age, mean (95% CI)	33.3 (29.2–37.3)	40.4 (31.2–49.5)
BMI		
Mean (95% CI)	24.7 (23.6–25.3)*	26.85 (25.3–28.3)
Elevated (>25), *n* (%)	22 (41.5)	11 (73.3)
Smoking, *n* (%)	22 (41.5)	7 (46.6)

Statistically significant differences are marked with asterisk (*).

**Table 2:** Endoscopic and histologic data in GERD patients and healthy controls.

	GERD patients (*n* = 53)	Healthy controls (*n* = 15)
	Esophagus	

Endoscopy, *n* (%)		
Esophagitis grade		
0	20 (37.7)	15 (100)
A	15 (28.3)	0
B	13 (24.5)	0
C	3 (5.6)	0
D	0	0
Barrett's	3 (5.6)	0
Hernia	18 (33.9)	4 (26.6)
Presence of bile	4 (7.5)	0
Histology, *n* (%)		
Esophagitis	45* (84.9)	8 (53.3)
Barrett's	3(5.6)	0
Dysplasia	0	0

	Stomach	

Endoscopy, *n* (%)		
*Gastritis *		
No	11 (20.7)	10 (66.6)
Mild	30* (56.6)	5 (33.3)
Erosive	12* (22.6)	0
*Presence of bile *	27* (50.9)	3 (20)
Histology, *n* (%)		
*Gastritis *	50 (94.3)	10 (66.6)
*Atrophy *	17* (32)	1 (6.6)
*H. pylori (yes) *	16 (30.2)	3 (20)
*Cardiac metaplasia *	5 (9.4)	1 (6.6)
*Antrum metaplasia *	12* (22.6)	0
*Dysplasia *	0	0

Statistically significant differences are marked with asterisk (*).

**Table 3:** Demographic and clinical data in BRI-positive and BRI-negative GERD patients.

	BRI-positive (*n* = 25)	BRI-negative (*n* = 28)
Gender, *n* (M/F)	16/9	26/2
Age, mean (95% CI)	37.96 (30.6–45.2)*	29.18 (25.3–33)
BMI		
Mean (95% CI)	24.2 (22.8–25.6)	24.75 (23.7–25.8)
Elevated (>25), *n* (%)	10 (40)	12 (42.8)
Smoking, *n* (%)	10 (40)	11 (39.3)

Statistically significant differences are marked with asterisk (*).

**Table 4:** Endoscopic and histologic data in BRI-positive and BRI-negative GERD patients.

	BRI-positive (*n* = 25)	BRI-negative (*n* = 28)
	Esophagus	

Endoscopy, *n* (%)		
Esophagitis Grade		
0	7 (28)	13 (46.4)
A	5 (20)	10 (35.7)
B	8 (32)	5 (17.8)
C	3 (12)	0
D	0	0
Barrett's	3 (12)	0
Hernia	10 (40)	8 (28.5)
Presence of bile	2 (8)	2 (7)
Histology, *n* (%)		
Esophagitis	22 (88)	23 (82.1)
Barrett's	3 (12)	0
Dysplasia	0	0

	Stomach	

Endoscopy, *n* (%)		
*Gastritis *		
No	3 (12)	8 (28.5)
Mild	15 (60)	15 (53.5)
Erosive	7 (28)	5 (17.8)
*Presence of bile *	13 (52)	14 (0.5)
Histology, *n* (%)		
*Gastritis *	24 (96)	26 (92.8)
*Atrophy *	11 (44)	6 (21.4)
*H. pylori *	3* (12)	13 (46.4)
*Cardiac metaplasia *	5 (20)	1 (3.5)
*Antrum metaplasia *	9* (36)	3 (10.7)
*Dysplasia *	0	0

Statistically significant differences are marked with asterisk (*).

**Table 5:** Immunohistochemical study of the p53 expression in esophageal and gastric mucosa of GERD patients and HCs.

	GERD (*n* = 53)				Healthy controls (*n* = 15)			
Grade of p53 staining(*n*)	Esophagus		Stomach		Esophagus		Stomach	
	BRI (+) (*n* = 25)	BRI (−) (*n* = 28)	BRI (+) (*n* = 25)	BRI (−) (*n* = 28)	BRI (+) (*n* = 2)	BRI (−) (*n* = 13)	BRI (+) (*n* = 2)	BRI (−) (*n* = 13)
0	15	21	21	26	2	10	2	11
1	9	7	4	2	—	3	—	2
2	—	—	—	—	—	—	—	—
3	1	—	—	—	—	—	—	—
4	—	—	—	—	—	—	—	—
5	—	—	—	—	—	—	—	—

According to the percentages of the stained epithelial cells, the immunohistological sections were scored and graded as follows: 0 = absence of staining, 1 = staining up to 5%, 2 = staining>5%–10%, 3 = staining>10%–25%, 4 = staining>25%–50%, and 5 = staining>51%.

**Table 6:** Immunohistochemical study of the Ki-67 expression in esophageal and gastric mucosa of GERD patients and HCs.

	GERD (*n* = 53)				Healthy controls (*n* = 15)			
Grade of Ki-67 staining (*n*)	Esophagus		Stomach		Esophagus		Stomach	
	BRI (+) (*n* = 25)	BRI (−) (*n* = 28)	BRI (+) (*n* = 25)	BRI (−) (*n* = 28)	BRI (+) (*n* = 2)	BRI (−) (*n* = 13)	BRI (+) (*n* = 2)	BRI (−) (*n* = 13)
0	—	—	—	—	—	—	—	—
1	17	17	11	17	1	9	1	9
2	3	3	4	—	1	3	—	3
3	2	5	3	5	—	1	—	1
4	3	2	4	3	—	—	1	—
5	—	1	3	3	—	—	—	—

According to the percentages of the stained epithelial cells, the immunohistological sections were scored and graded as follows: 0 = absence of staining, 1 = staining up to 5%, 2 = staining>5%–10%, 3 = staining>10%–25%,4 = staining>25%–50%, and 5 = staining>51%.
